# Physical, psychological and occupational consequences of job burnout: A systematic review of prospective studies

**DOI:** 10.1371/journal.pone.0185781

**Published:** 2017-10-04

**Authors:** Denise Albieri Jodas Salvagioni, Francine Nesello Melanda, Arthur Eumann Mesas, Alberto Durán González, Flávia Lopes Gabani, Selma Maffei de Andrade

**Affiliations:** 1 Department of Nursing, Instituto Federal do Paraná, Londrina, Paraná, Brazil; 2 Department of Pathological Sciences, Universidade Estadual de Londrina, Paraná, Brazil; 3 Department of Public Health, Universidade Estadual de Londrina, Paraná, Brazil; 4 Department of Nursing, Universidade Estadual de Londrina, Paraná, Brazil; TNO, NETHERLANDS

## Abstract

Burnout is a syndrome that results from chronic stress at work, with several consequences to workers’ well-being and health. This systematic review aimed to summarize the evidence of the physical, psychological and occupational consequences of job burnout in prospective studies. The PubMed, Science Direct, PsycInfo, SciELO, LILACS and Web of Science databases were searched without language or date restrictions. The Transparent Reporting of Systematic Reviews and Meta-Analyses guidelines were followed. Prospective studies that analyzed burnout as the exposure condition were included. Among the 993 articles initially identified, 61 fulfilled the inclusion criteria, and 36 were analyzed because they met three criteria that must be followed in prospective studies. Burnout was a significant predictor of the following physical consequences: hypercholesterolemia, type 2 diabetes, coronary heart disease, hospitalization due to cardiovascular disorder, musculoskeletal pain, changes in pain experiences, prolonged fatigue, headaches, gastrointestinal issues, respiratory problems, severe injuries and mortality below the age of 45 years. The psychological effects were insomnia, depressive symptoms, use of psychotropic and antidepressant medications, hospitalization for mental disorders and psychological ill-health symptoms. Job dissatisfaction, absenteeism, new disability pension, job demands, job resources and presenteeism were identified as professional outcomes. Conflicting findings were observed. In conclusion, several prospective and high-quality studies showed physical, psychological and occupational consequences of job burnout. The individual and social impacts of burnout highlight the need for preventive interventions and early identification of this health condition in the work environment.

## Introduction

Working conditions have a well-known impact, either positive or negative, on employees’ health [1]. Adverse working conditions may lead to job burnout, a syndrome resulting from chronic stress at work that is characterized by overwhelming exhaustion, negative attitudes or a lack of commitment with clients and dissatisfaction with job performance. This process may lead to undesirable consequences for workers, their families, the work environment and the organizations [2]. From the psychosocial perspective, the following three dimensions of burnout have been described: a) emotional exhaustion, characterized by emotional depletion and loss of energy; b) depersonalization or cynicism, also described as dehumanization, detachment from work and clients and emotional hardening; and c) reduced personal accomplishment or inefficacy, that is, a feeling of personal or professional inadequacy as well as reduced productivity and coping skills [3, 4].

Cross-sectional studies have shown associations between burnout and some health problems, such as increased alcohol consumption [5], sleep disorders [6] depression [7], sedentarism, obesity [8] and musculoskeletal pain [9]. However, well-conducted prospective studies are more appropriate for investigating the possible consequences of this syndrome, because these types of studies enable the identification of the temporal relationship between the exposure (burnout syndrome) and the outcomes (consequences).

We found only two systematic reviews that have investigated burnout and its possible consequences in working populations. One selected only studies published in English between 1988 and 2008, examined only job satisfaction and turnover intention among North-American psychotherapists and included only studies with a cross-sectional design [10]. The other focused on nurses and investigated the relationships of burnout, job satisfaction and general health in findings from 70 studies published in English between 1990 and 2012; the majority of these (68 studies) were also cross-sectional studies [11].

Therefore, this systematic review aimed to summarize the evidence of the physical, psychological and occupational consequences of job burnout in prospective studies.

## Materials and methods

This study is a systematic review that followed the guidelines of the Transparent Reporting of Systematic Reviews and Meta-Analyses–S1 Appendix [12] (PROSPERO Register: CRD42015028047). We searched the PubMed (U.S. National Library of Medicine), Science Direct (Elsevier), PsycInfo (American Psychological Association), SciELO (Scientific Electronic Library Online), LILACS (Literature in the Health Sciences in Latin America and the Caribbean) and Web of Science databases through May 31, 2017, without restrictions on language or year of publication.

### Search strategy

The following terms were used to identify publications on burnout with a prospective design: “burnout”, “longitudinal”, “prospective”, “cohort”, “case control”, “case-control”, “follow-up”, and “follow up”. These terms were combined with Boolean operators according to the rules of each database (S2 Appendix). To complement the database searches, we reviewed all the references of the selected articles and those of review articles.

### Inclusion and exclusion criteria

The inclusion criteria were as follows: the study was an original study published in a journal with an editorial board and peer review; and the study was a prospective study investigating burnout as the exposure (independent variable) for the occurrence of physical, psychological or occupational consequences (dependent variables).

We excluded studies of non-working populations (i.e., studies with twins, patients and students, including medical, sports and high school students), editorials, commentaries, letters to editors, abstracts, literature reviews, qualitative studies, studies that reported only a cross-sectional analysis, trials, studies that reported research method or instrument validation, and follow-up studies that did not have a comparison group (unexposed to burnout) or treated burnout as an outcome (dependent variable).

### Selection and extraction of the articles

The selection of the studies was performed independently by two of the authors using the following three steps: (a) analyzing the articles’ titles, (b) reading the abstracts, and (c) reading the full texts. At each step, if there were divergences, a third author was asked to judge, and the final decision was made by consensus or majority. After selection of the studies, the data of interest were registered in a standardized spreadsheet.

### Evaluating the methodological quality of the studies

**Fig 1:**
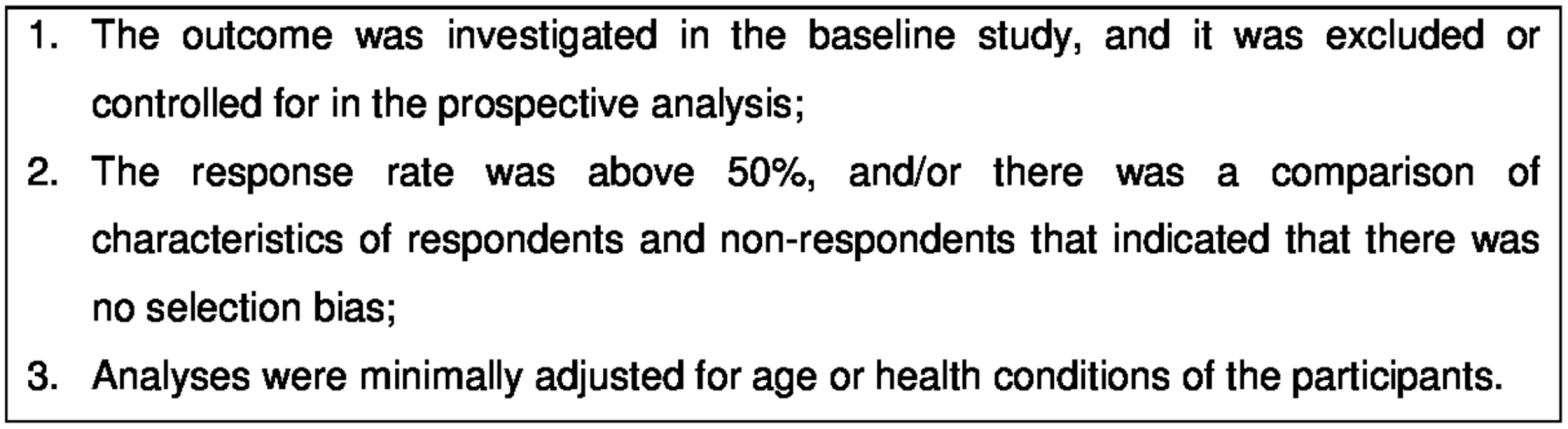
Evaluation criteria of the methodological quality of the studies.

Although several tools for evaluating the susceptibility to bias in observational epidemiological studies are available, no consensus has been reached on a gold standard for this purpose [13, 14]. Moreover, many of these tools have been criticized either because their validity and reliability have not been reported [15, 16] or because of low agreement between reviewers and authors [17] or between reviewers [18]. Therefore, we analyzed several of these tools [19–22] as well as another tool related to adequate reporting of observational epidemiological studies [23]. Based on the conclusions of that analysis, we decided to follow the recommendations of epidemiology experts [24, 25] for conducting sound and reliable prospective studies and only considered studies that met three central methodological criteria (Fig 1) for internal validity. These criteria were related to the selection of participants (ensuring that those with the outcome already present at the baseline study were excluded), the attrition from the baseline study to the end of follow-up and adjustments for the main confounders.

The first criterion was chosen because, in cohort studies, the exposed and unexposed groups are followed in order to compare the incidence (new cases) of the outcome in both groups; therefore, cases already present at baseline should be excluded [25]. However, we included studies that controlled for the outcome in the analysis of follow-up time. Additionally, changes in the levels of the outcome over time (from baseline to the end of the follow-up time) were considered incident events.

The response rate or loss to follow-up is another relevant aspect to be considered in epidemiological studies, as non-respondents or those lost to follow-up in a cohort study may differ in several characteristics from those who respond or are successfully observed during the time in which the study is conducted. These differences include whether the respondents were exposed to the hypothesized risk factor and whether they have a higher/lower risk of presenting the outcome at the end of the follow-up period [26]. Therefore, the second criterion was chosen to prevent the inclusion of studies that were susceptible to selection bias [24, 26].

As we investigated consequences of burnout, the third criterion was chosen because age and/or health conditions are important factors that may confound the associations [24]. Therefore, only studies that reported minimal control for age or for health conditions were analyzed.

### Data organization and presentation

The results are presented in tables according to the nature of the consequence: physical, psychological or occupational. When two types of consequences were reported by the same study, they were presented twice in their respective tables. The characteristics of the selected studies (authors, year of publication, country, cohort’s name, working population, follow-up period, burnout instrument, burnout measure, type of dependent variable and outcome measure) are presented in alphabetical order by the first authors’ surnames. The consequences were put in order according to their type and subtype (e.g., organic system), and the tables describe the final sample, the dependent variable (investigated consequence), the main significant and non-significant findings, and the control variables used in the analyses.

## Results

### Search for and selection of studies

**Fig 2:**
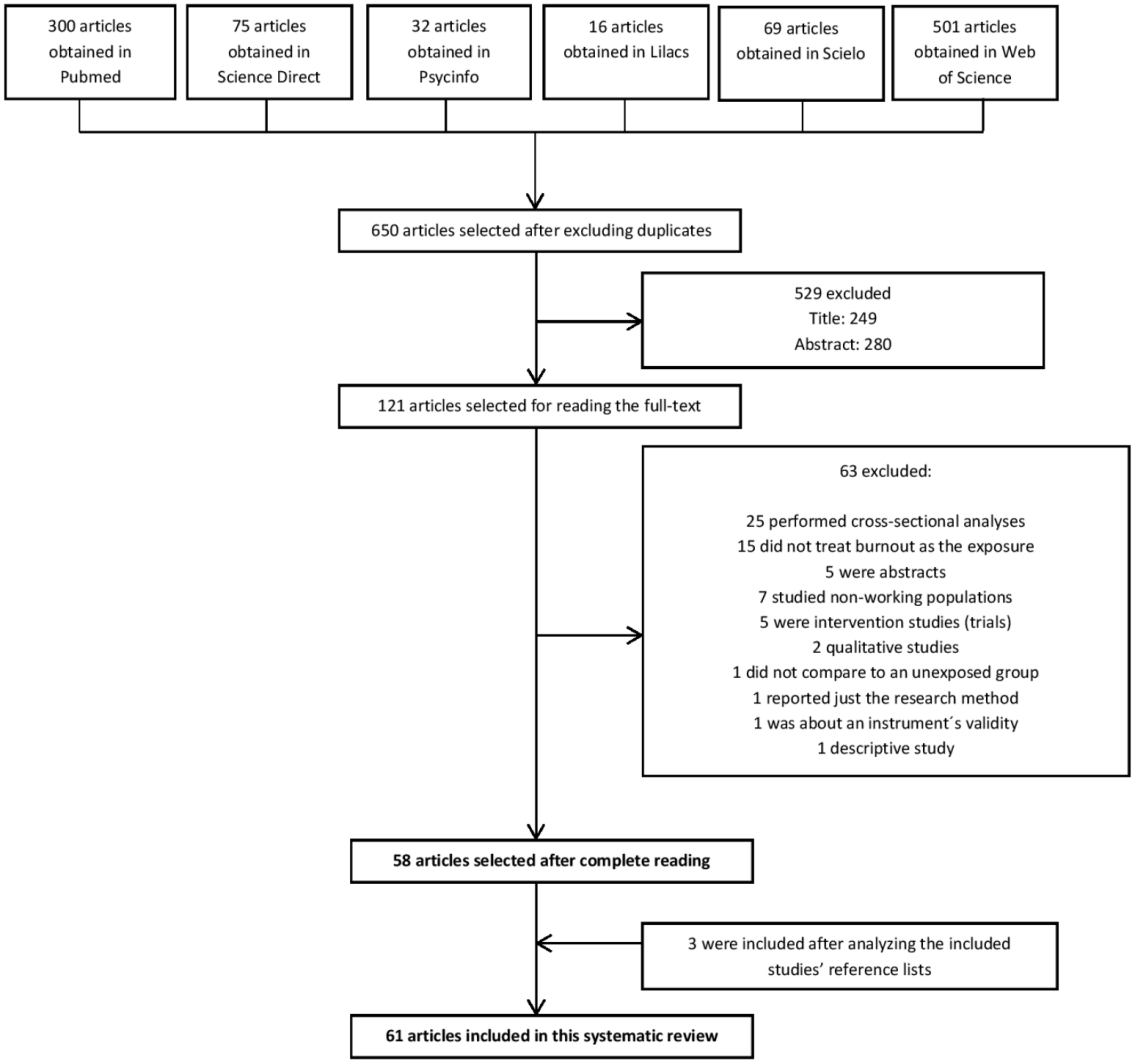
Flow diagram of the identification and selection of studies.

We identified 993 articles in the databases that were searched; 343 were duplicated. After reading the titles and abstracts, 529 were excluded. Among the 121 remaining articles, we excluded 63 after reading the full text. We then added three studies listed in the references of the selected studies, resulting in a final sample of 61 articles (Fig 2).

### Methodological quality evaluation

Among the 61 selected studies [27–87], 36 met all three methodological quality criteria (S3 Appendix). The main characteristics of the articles that did not meet our predefined quality criteria can be viewed in S4 Appendix.

### Characteristics of the included studies

From here on, only the characteristics and results of the 36 studies that met all three quality criteria will be presented (Table 1) and discussed. All studies were prospective cohorts; 12 reported physical consequences, 10 reported psychological consequences and 12 reported occupational consequences. Two articles analyzed physical and psychological consequences.

**Table 1:** Characteristics of the 36 articles included in this systematic review.

AUTHORS, YEAR COUNTRY	COHORT NAME	WORKING POPULATION	FOLLOW-UP PERIOD	BURNOUT INVENTORY	BURNOUT MEASURE	DEPENDENT VARIABLE	OUTCOME MEASURE
Ahola et al., 2007 -Finland [27]	Not identified	Dentists	3 years	Maslach Burnout Inventory (MBI)	A sum score, in which exhaustion, cynicism, and lack of professional efficacy have different weights (0.4×exhaustion+0.3× cynicism+0.3×lack of professional efficacy). Burnout was categorized as follows: no burnout (scores 0–1.49), mild burnout (scores 1.50–3.49), and severe burnout (scores 3.50–6)	Depressive symptoms	Beck Depression Inventory (BDI)
Ahola et al., 2009 -Finland [28]	Health 2000 Study	Employees randomly selected	4 years	MBI	A sum score, in which exhaustion, cynicism, and lack of professional efficacy have different weights (0.4×exhaustion+0.3× cynicism+0.3×lack of professional efficacy). Burnout was categorized as follows: no burnout (scores 0–1.49), mild burnout (scores 1.50–3.49), and severe burnout (scores 3.50–6)	New disability pension	Records of the Social Insurance Institution of Finland and the Finnish Centre for Pensions
Ahola et al., 2009 -Finland [29]	Still Working Cohort Stud	Forest industry employees	8 years	MBI	A sum score, in which exhaustion, cynicism, and lack of professional efficacy have different weights (0.4×exhaustion+0.3× cynicism+0.3× lack of professional efficacy). Burnout was categorized as follows: no burnout (scores 0–1.49), mild burnout (scores 1.50–3.49), and severe burnout (scores 3.50–6)	New disability pension	Records of the Social Insurance Institution of Finland and the Finnish Centre for Pensions
Ahola et al., 2010 -Finland [30]	Still Working Cohort Study	Forest industry employees	10 years	MBI	A sum score, in which exhaustion, cynicism, and lack of professional efficacy have different weights (0.4×exhaustion+0.3× cynicism+0.3× lack of professional efficacy)	Mortality	Death certificates from the National Mortality Register
Ahola et al., 2013 -Finland [31]	Still Working Cohort Study	Forest industry employees	8 years	MBI	A sum score, in which exhaustion, cynicism, and lack of professional efficacy have different weights (0.4×exhaustion+0.3× cynicism+0.3× lack of professional efficacy).Burnout was categorized as follows: no burnout (sum score 0 to 1.49) and burnout (sum score 1.50 to 6)	Severe Injuries	Data on deaths from the National Mortality Register. Data on hospital admissions from the National Hospital Discharge Register
Appels, Schouten, 1991 Netherlands [32]	Rotterdam Civil Servants Study	Male employees who participated in a voluntary health checkup	4.2 years	No scale. Burnout was verified by the questions: Have you ever been burned out? “Yes”, “?” or “no”	Dichotomous variable (ever burned out, never burned out)	Coronary Heart Disease	Medical diagnosis of cardiac problems registered in a central system. For employees who had left their jobs, a questionnaire during follow-up (self-reported coronary disease, which was then checked by a doctor); death certificates
Armon et al., 2008 -Israel [33]	Tel Aviv Sourasky Medical Center	Apparently healthy employees attending the Center for Periodic Health Examinations	1.5 years	Shirom-Melamed Burnout Measure (SMBM)	Dichotomous variable (using the 80th percentile as the cut-off point)	Insomnia	A slightly modified Brief Athens Insomnia Scale (AIS-5)
Armon et al., 2008 -Israel [34]	Tel Aviv Sourasky Medical Center	Apparently healthy employees attendingthe Center for Periodic Health Examinations	1.5 years	SMBM	Continuous variable (mean of burnout score)	Obesity—Body Mass Index (BMI), Waist Circumference (WC), Waist-to-Hip Ratio (WHR)	BMI (kg/m^2^), WHR (in centimeters), and WC (in centimeters) were measured by a nurse
Armon, 2009 -Israel [35]	Tel Aviv Sourasky Medical Center	Apparently healthy employees attending the Center for Periodic Health Examinations	1.5 years	SMBM	Continuous variable (mean of burnout score)	Changes in levels of insomnia	A slightly modified Brief Athens Insomnia Scale (AIS-5)
Armon et al., 2010 -Israel [36]	Tel Aviv Sourasky Medical Center	Apparently healthy employees attending the Center for Periodic Health Examinations	3 years(T1-T2: 18 months,T2-T3: 17 months)	SMBM	Continuous variable (mean of burnout score)	Musculoskeletal Pain	Self-reported neck pain, pain in the shoulder region, or lower back pain over the last 12 months
Armon et al., 2014 -Israel [37]	Tel Aviv Sourasky Medical Center	Apparently healthy employees attending the Center for Periodic Health Examinations	1.5 years	SMBM	Continuous variable (mean of burnout score)	Depressive symptoms	Patient Health Questionnaire (PHQ-8)
Bianchi et al., 2015 -France [38]	Not identified	School teachers	21 months (mean)	MBI	Continuous variable (mean of burnout score)	Depressive symptoms	9-item depression module of the Patient Health Questionnaire (PHQ-9)
Borritz et al., 2006 -Denmark [39]	PUMA Study	Employees (seven different organizations in the human service sector)	3 years (mean)	Copenhagen Burnout Inventory (CBI)	The scores were divided into the following three categories: low (25th-percentile), medium (the 25th-to 75th-percentile), and high (the 75th-percentile)	Sickness absence days and sickness absence spells	Self-reported sickness absence (days and spells) in the previous 12 months
Borritz et al., 2010 -Denmark [40]	PUMA Study	Employees (five different organizations in the human service sector)	1.5 years	CBI	The scores were divided into three categories: low (25th-percentile), medium (the 25th-to 75th-percentile), and high (the 75th-percentile)	Long-Term Sickness Absence (>2 weeks)	Data from a Danish national register of social transfer payments database (DREAM)
De Beer et al., 2016 -South Africa [44]	Not identified	Employees from the financial services sector	3 years (annually)	Burnout was measured by exhaustion(four items) and cynicism (four items)	Continuous variable (mean of burnout score)	Psychological Ill-Health Symptoms	7-item scale of the South African Employee Health and Wellness Survey (SAEHWS)
Demerouti et al.,2009 -Netherlands [45]	Not identified	Staff nurses in general hospitals	1.5 year	MBI	Continuous variable (mean of emotional exhaustion and depersonalization)	Job demands and Presenteeism	Job demands: 5-point Likert Furka scale. Presenteeism: “Has it happened over the previous 12 months that you have gone to work despite feeling sick?”
Figueiredo-Ferraz et al.,2012 -Spain [47]	Not identified	Nurses of three hospitals	1 year	MBI	Continuous variable (mean of emotional exhaustion, depersonalization and professional efficacy)	Job satisfaction	11-item Satisfaction Questionnaire S20/23
Grossi et al., 2009 -Sweden [48]	Not identified	Women employees randomly selected from the general population living in Stockholm County	1 year	SMBM	Continuous variable (sum of emotional exhaustion/physical fatigue and cognitive difficulties scores)	Changes in pain experiences	Pain variables were measured using the Pain Questionnaire. Pain was defined as pain of at least 1 month in duration experienced during the past 3 months in specific sites (e.g., back)
Hallsten et al., 2011 -Sweden [51]	HAKuL Study	Public employees (nurses, homecare workers, teachers, clerical officers and childcare employees)	1 year	MBI	Exhaustion and cynicism items were retained to calculate the core MBI-GS scores. The scores were divided into the following three categories: low ExCy (25th-percentile), medium ExCy (25th-to 75th-percentile), and high ExCy (75th-percentile)	Long‑term sickness absence(>60 consecutive days)	For each participant, the dates of the first and last day of each spell of sick leave were available in the employers’ registers on absences from work
Jansson-Fröjmark, Lindblom, 2010 -Sweden [55]	Not identified	Employees randomly selected from the general population	1 year	MBI	Burnout was defined for the three subscales as follows: a score of 4.6 or above on the Emotional Exhaustion subscale (low/high), 3.5 or above on the Cynicism subscale (low/ high), and 3.6 or below on the Professional Efficacy subscale (low/high)	Incidence and persistence of insomnia	Basic Nordic Sleep Questionnaire and the Uppsala Sleep Inventory
Kim et al., 2011 -USA [58]	Not identified	Social workers	3 years (annually)	MBI	Continuous variable (mean of burnout score)	Sleep disturbances, headaches, respiratory infections and gastrointestinal infections.	Physical Health Questionnaire (PHQ)
Kitaoka-Higashiguchi et al, 2009 -Japan [59]	Not identified	Male middle managers working for a manufacturing company	4–5 years	MBI (Japanese Version)	For exhaustion and cynicism, the cut-off point was set between the upper third and the lower two-thirds, and for professional efficacy, the cut-off point was set between the higher two-thirds and the lower third. Subjects with intense exhaustion and either a high level of cynicism or a low level of professional efficacy, or both, were considered to have burnout	Risk factors for arteriosclerotic disease	Anthropometric measurements and laboratory test
Leiter et al., 2013 -Finland [60]	Still Working Cohort Study	Forest industry employees	12 years (T1-T2: 4 years, T2-T3: 8 years)	MBI	Continuous variable (mean of emotional exhaustion and depersonalization; professional efficacy and inconsistency among the dimensions)	Use of psychotropicand antidepressant medications	Data from the National Prescription Register (number of prescriptions)
Leone et al., 2009 -Netherlands [61]	MaastrichtCohort Study on Fatigue at Work	Employees from 45 different companies and organizations	4 years (12-, 24-, and 48-month)	MBI (Dutch Version)	Burnout cases were defined as having a score higher than the 75th percentile on exhaustion and a score higher than the 75th percentile on cynicism *or* a score lower than the 25th percentile on professional efficacy	Prolonged fatigue	20-item Checklist Individual Strength (CIS)
Lizano, Barak, 2015 -USA [62]	Not identified	Workers of child welfare department	1 year (two points in time with a 6-month interval)	MBI	Continuous variable (mean of emotional exhaustion and depersonalization)	Job satisfaction	4-item scale of Quality of Employment Survey
Madsen et al., 2015 -Denmark [63]	PUMA Study	Human service workers	6 years (every 3 years)	CBI	The scores were divided into three categories: low (≤25), intermediate (25<score≤50) and high (>50) burnout	Antidepressant treatment	Data from the Danish National Prescription Registry (number of prescriptions)
Melamed et al., 2006 -Israel [65]	Not identified	Employees from seven work organizations (two metal factories, two pharmaceutical companies, a textile factory, a food factory and a school)	3–5 years (mean 3.6 years)	SMBM	Dichotomous variable (high burnout—above the mean score) and continuous variable (mean of score burnout)	Type 2 Diabetes	Based on self-reports of diagnosed and treated type 2 diabetes
Melamed, 2009 -Israel [66]	Not identified	Employees from seven work organizations (two metal factories, two pharmaceutical companies, a textile factory, a food factory and a school)	3–5 years (mean 3.6 years)	SMBM	Continuous variable (mean of burnout score)	Musculoskeletal pain	Reported neck pain, pain in the shoulder region, or lower back pain over the last 12 months
Roelen et al., 2015 -Netherlands [68]	Not identified	Employees who participated in an occupational health survey	1 year	MBI	Continuous variable (mean of exhaustion, cynicism and burnout scores–calculated by summing the scores of exhaustion and cynicism)	Long-term sickness absence–LTSA (≥42 consecutive days)	LTSA was medically certified with a diagnostic code of the ICD-10, which was recorded in the occupational health service register
Schaufeli et al., 2009 -Netherlands [71]	Not identified	Managers and executives of a Dutch telecom company	1 year	MBI	Continuous variable (mean of exhaustion and cynicism)	Absence duration (number of sick-leave days betweenT1 and T2)	Sickness absence records of the employees filed in the database of the company’s occupational health service
Shirom et al., 2013 -Israel [74]	Tel Aviv Sourasky Medical Center	Apparently healthy employees attending the Center for Periodic Health Examinations	2.3 years	SMBM	Continuous variable (mean of burnout score)	Hyperlipidemia	Respondents self-reporting that a physician told them that they had hyperlipidemia
Toker et al., 2012 -Israel [77]	Tel Aviv Sourasky Medical Center	Apparently healthy employees attending the Center for Periodic Health Examinations	7 years (3.6 years on average)	SMBM	Continuous variable (mean of burnout score) and burnout as a dichotomous variable (high—upper most quintile; low burnout—otherwise)	Coronary Heart Disease (CHD)	Participants’ self-reports of medically diagnosed CHD
Toker, Biron, 2012 -Israel [78]	Tel Aviv Sourasky Medical Center	Apparently healthy employees attending the Center for Periodic Health Examinations	3 waves between 2003 and 2009	SMBM	Continuous variable (mean of burnout score)	Depressive symptoms	Personal Health Questionnaire (PHQ-8)
Toppinen-Tanner et al., 2005 -Finland [79]	Still Working Cohort Study	Forest industry employees	1.8 years	MBI	A sum score, in which exhaustion, cynicism, and lack of professional efficacy have different weights (0.4×exhaustion+0.3× cynicism+0.3× reduced of professional efficacy). The 3 individual components of burnout were analyzed separately and trichotomized as follows: low, medium, and high (divided by terciles)	Sick-Leave Absences(≥3 days absence episodes, medically certified)	Company registers (number of episodes and the total number of sick-leave days)
Toppinen-Tanner et al., 2009 -Finland [80]	Still Working Cohort Study	Forest industry employees	10 years	MBI	A sum score, in which exhaustion, cynicism, and lack of professional efficacy have different weights (0.4×exhaustion+0.3× cynicism+0.3× lack of professional efficacy)	Hospitalization for cardiovascular, musculoskeletal and mental disorders	The data was derived from the National Hospital Discharge Register
Wang et al., 2016 -China [83]	Not identified	Workers at companies specializing in software development, electronic engineering, and agricultural products	1 year (two points in time with a 6- month interval)	MBI	Continuous variable (mean of burnout score)	Job demands and job resources	Job Content Questionnaire), SWING Questionnaire, Job Diagnostic Survey

The studies were mainly conducted in Europe, particularly in Nordic countries (21 studies). The only other regions of the world in which the studies were conducted were Asia (Israel, China and Japan), South Africa and the United States. Several occupations were investigated, such as dentists, forest industry employees, nurses, teachers, human service workers and financial services′ employees. The Maslach Burnout Inventory (MBI) and the Shirom-Melamed Burnout Measure (SMBM) were the primary tools used to investigate burnout. Most authors treated burnout as a continuous variable, while some authors treated burnout as dichotomous and others divided it into three categories. The number of participants varied from 133 [62] to 10,062 [31]. Follow-up time ranged from one year for several outcomes [47, 48, 51, 55, 62, 68, 83] to 12 years for the outcome use of psychotropic and antidepressant medications [60]. With the exception of one study [32], all studies were published between the years 2005 and 2016. Only one study was not reported in English [47].

### Physical consequences

The most frequently investigated physical outcomes were cardiovascular diseases (coronary heart disease (CHD) and hospitalization for cardiovascular diseases) and risk factors for these diseases (obesity, hyperlipidemia, type 2 diabetes, large waist circumference (WC), high body mass index (BMI), metabolic syndrome, hypertension, high triglycerides, low HDL cholesterol, high LDL cholesterol, and impaired fasting glucose) (Table 2).

**Table 2:** Main findings of longitudinal studies of the physical consequences of burnout.

AUTHORS, YEAR	N (FINAL SAMPLE)	DEPENDENT VARIABLE	MAIN FINDINGS		
			NOT SIGNIFICANT	SIGNIFICANT	CONTROL VARIABLES
Armon et al., 2008 [34]	1,064	Obesity	Waist-to-hip ratio: r = 0.02;Waist circumference: r = 0.02;Body Mass Index: r = 0.03	-	Depressive symptomatology, physical exercise and age
Shirom et al., 2013 [74]	3,337	Hyperlipidemia	B = 0.04 Wald Test = 0.16	-	Age, gender, obesity, education, smoking, financial strain, time lag T1-T2 (days) and physical exercise
Melamed et al., 2006 [65]	677	Type 2 Diabetes	-	OR = 1.84 (1.19–2.85)**	Age, sex, body mass index, smoking, alcohol use, leisure time physical activity, initial job category, and follow-up duration
Kitaoka-Higashiguchi et al., 2009 [59]	383	Risk factors for arteriosclerotic disease	**Large waist circumference** (WC ≥85 cm)OR = 1.46 (0.49–4.38)**High body mass index** (BMI ≥25)OR = 1.97 (0.49–7.92)**Metabolic syndrome**OR = 1.17 (0.33–4.15)**Hypertension** (blood pressure ≥140/90 mmHg)OR = 0.67 (0.25–1.77)**High triglycerides** (triglycerides ≥150 mg/dl)OR = 1.19 (0.43–3.30)**Low HDL cholesterol** (HDL cholesterol <40 mg/dl)OR = 0.83 (0.09–7.50)**High LDL cholesterol** (LDL cholesterol ≥140 mg/dl)OR = 1.34 (0.49–3.69)**Impaired fasting glucose** (fasting blood sugar ≥110 mg/dl)OR = 0.58 (0.12–2.71)	**Hypercholesterolemia** (total cholesterol ≥220 mg/dl)OR = 2.78 (1.20–6.46)	Age, alcohol consumption, smoking, and physical activity
Appels, Schouten, 1991 [32]	3,210	Coronary Heart Disease	-	**Model 1**: RR = 2.11 (1.28–3.48)**Model 2: RR = 2.26 (1.37–3.72)**Model 3: RR = 2.25 (1.37–3.69)**Model 4: RR = 2.22 (1.35–3.66)**Model 5: RR = 2.16 (1.31–3.55)**	**Model 1**: age**Model 2**: cholesterol**Model 3**: systolic blood pressure**Model 4**: diastolic blood pressure**Model 5**: smoking
Toker et al., 2012 [77]	8,838	Coronary Heart Disease	**-**	**BurnoutModel 1**: HR = 1.31 (1.03–1.66)*Model 2: HR = 1.41 (1.08–1.85)*High BurnoutModel 1: HR = 1.70 (1.05–2.75)*Model 2: HR = 1.79 (1.05–3.04)*	**Model 1**: age, sex, family history of CHD, cigarette smoking, education years, LDL, glucose, body mass index, systolic blood pressure, and exercise hours per week. **Model 2**: age, sex, smoking status, family history, depression and workload
Toppinen-Tanner et al., 2009 [80]	7,897	Hospitalization for cardiovascular and musculoskeletal disorders	**Hospitalization for musculoskeletal disorders** HR = 1.05 (0.98–1.13)	**Hospitalization for cardiovascular disorders** HR = 1.10 (1.02–1.19)	**Hospitalization for cardiovascular disorders**: age, sex, occupational status, physical environment, and use of medication for hypertension or diabetes.**Hospitalization for musculoskeletal disorders**: age, sex, occupational status, and physical environment at baseline
Armon et al., 2010 [36]	1,068	Musculoskeletal Pain	-	OR = 2.09 (1.07–4.10)*	Depressive symptomatology, body mass index, gender, educational level, and age
Melamed,2009 [66]	650	Musculoskeletal Pain	-	**Burnout mean score** OR = 1.67 (1.14–1.87)**High burnout level OR = 2.45 (1.35–4.45)	Age, gender, body mass index, smoking, leisure time physical activity, and blue-collar work
Grossi et al., 2009 [48]	2,300	Changes in pain experiences	**Headache** Burnout at T1: OR = 0.99 (0.71–1.37) Burnout changes (T2-T1): OR = 1.34 (0.98–1.82) **Pain in the entire body** Burnout at T1: OR = 1.87 (0.98–3.54) Burnout changes (T2-T1): OR = 1.77 (0.95–3.28) **Pain intensity** Burnout at T1: β = -0,08 Burnout changes (T2-T1): β = 0.01 **Pain frequency** Burnout at T1: β = 0.07 Burnout changes (T2-T1): β = 0.06	**Overall pain** Burnout at T1: OR = 1.70 (1.34–2.16)** Burnout changes (T2-T1): OR = 1.63 (1.31–2.03)** **Neck-shoulder pain** Burnout at T1: OR = 1.64 (1.30–2.07)** Burnout changes (T2-T1): OR = 1.63 (1.31–2.04)** **Back pain** Burnout at T1: OR = 1.49 (1.19 = 1,87)** Burnout changes (T2-T1): OR = 1.49 (1.20–1.85)** **Pain-related disability** Burnout at T1: β = 0.23** Burnout changes (T2-T1): β = 0.19**	Sociodemographic (e.g., age, marriage, and education), work characteristics, smoking, psychological distress, physical health and basal pain parameters
Leone et al., 2009 [61]	5,328	Prolonged fatigue	-	HR = 1.33 (1.16–1.53)	Fatigue at baseline, age, gender, education and absenteeism at baseline
Kim et al., 2011 [58]	146	Headaches, respiratory infections, and gastrointestinal problems	-	**Headaches** β = 0.23* **Gastrointestinal problems** β = 0.20* **Respiratory infections** β = 0.19*	Age, gender, field tenure and annual salary
Ahola et al., 2013 [31]	10,062	Severe Injuries (transport accidents, falls, other external causes of accidental injury, exposure to the forces of nature and accidental exposure to unspecified factors)	-	HR = 1.18 (1.02–1.36)	Age, sex, marital status, and occupational status
Ahola et al., 2010 [30]	7,396	Mortality	**45 years or older** HR = 0.99 (0.84–1.17)	**Below 45 years of age** HR = 1.31 (1.04–1.66)	Gender, marital status, socioeconomic status, common risk factors for health and work ability

*p<0.05

**p<0.01

HR = hazard ratio OR = odds ratio RR = risk ratio β = standardized partial regression coefficients B = unstandardized partial regression coefficients r = intercorrelations

Burnout was a significant predictor of hypercholesterolemia (total cholesterol ≥220 mg/dl) [59] and type 2 diabetes [65], independently of confounding variables. Burnout was also associated with low HDL cholesterol in a model adjusted for age; however, this association lost significance when additionally adjusted for alcohol consumption, smoking and physical activity [59].

Two studies confirmed a higher incidence of CHD among those exposed to burnout [32, 77]. A significant association between burnout and hospitalizations due to cardiovascular diseases has also been observed in a cohort study of industrial employees that lasted 10 years [80].

Musculoskeletal disorders have been shown to be significantly associated with burnout. Increased levels of burnout during 18 months of follow-up were associated with an increased risk of developing musculoskeletal pain [36]. Workers with high burnout levels had more than twice the risk of developing musculoskeletal pain compared to those without burnout [66]. Burnout was also a risk factor for hospitalizations due to musculoskeletal disorders after adjusting for age and gender; however, this association lost significance after further adjustments by occupational status and physical environment at baseline [80].

One study found a relationship between burnout and changes in pain experiences. Burnout at T1 (baseline) or changes in burnout levels between T1 and T2 were important predictors of overall pain, neck-shoulder pain, back pain, and pain-related disability. However, pain in the entire body, pain intensity and pain frequency were not associated after adjustments [48]. Headache was an outcome of burnout investigated in two studies [48, 58], but a significant association was found in only one study [58]. Both studies differed in the types of populations, measures of headache and control variables used in the analyses. The study that yielded a significant association was conducted among social workers in the US and measured headache through a general health questionnaire (PHQ) that contained 3 items related to headache (during the six previous months), resulting in scores ranging from 3 to 21; thus, headache was treated as a continuous variable. The analyses were adjusted for age, gender, field tenure and annual salary [58]. The other study investigated a random sample of Swedish women. Headache was assessed with a specific questionnaire on pain (several items) and was defined as pain during at least one month over the past three months; adjustments for the association between burnout and headache (incidence or increased ratings of intensity or frequency) were made for sociodemographic variables, work characteristics, smoking, psychological distress, physical health and basal pain parameters [48].

Some studies investigated burnout as a risk factor for prolonged fatigue [61], gastrointestinal issues, respiratory problems [58], severe injuries [31] and mortality below the age of 45 years [30]. All these consequences were significantly associated with burnout. However, burnout was not a significant predictor of mortality among those 45–65 years old [30].

### Psychological consequences

Insomnia and depressive symptoms were the main investigated psychological consequences (Table 3). Studies of 1,356 and 3,235 apparently healthy employees attending a center for a health examination showed burnout as a significant predictor of new cases of insomnia [33] and an increase in insomnia levels, respectively [35]. In these two studies [33, 35], insomnia was assessed using a slightly modified version of the AIS-5, which is a 5-item tool that evaluates difficulties with sleep induction and maintenance by self-reporting with a total score ranging from 5 to 35. In one study, new cases of insomnia were defined dichotomously (yes/no) [33], whereas in the other study [35], insomnia was treated as a continuous variable (changes in the level of the total score). However, a study of 1,258 workers randomly selected from the general working population in Sweden that analyzed the dimensions of burnout (emotional exhaustion, cynicism, and professional efficacy) separately did not find an association with the incidence (new cases of insomnia) or persistence of insomnia (from the baseline study until one year later) [55]. In this study, the authors defined insomnia as self-reported problems with sleep three or more times per week during the past three months and difficulty in initiating or maintaining sleep for 30 minutes or more per night [55]. Similarly, a study of 146 social workers in the USA found that burnout was not a predictor of sleep disturbances [58]. Sleep disturbances in this study were assessed using the Physical Health Questionnaire–PHQ, which is a 4-item tool encompassing self-reported difficulty in falling asleep, awakenings during the night, nightmares and perception of a peaceful or undisturbed night’s sleep.

**Table 3:** Main findings of longitudinal studies of psychological consequences of burnout.

AUTHORS, YEAR	N (FINAL SAMPLE)	DEPENDENT VARIABLE	MAIN FINDINGS		
			NOT SIGNIFICANT	SIGNIFICANT	CONTROL VARIABLES
Armon et al., 2008 [33]	1,356	Insomnia	-	OR = 1.93 (1.45–2.58) B = 0,06* β = 0,05*	Depressive symptomatology, body mass index, age and gender
Armon, 2009 [35]	3,235	Changes in levels of insomnia	-	T1 burnout predicted T2 insomnia (β = 0.06)	Insomnia (T1), depression, body mass index, age, gender and follow-up duration
Jansson-Fröjmark, Lindblom, 2010 [55]	1,258	Incidence and persistence of insomnia	**Emotional exhaustion** HR = 1.61 (0.80–3.26) **Cynicism** HR = 1.37 (0.73–2.56) **Professional efficacy** HR = 0.62 (0.28–1.41)	-	Age, gender, anxiety and depression
Kim et al., 2011 [58]	146	Sleep disturbances	Burnout was not associated with significant increases in sleep disturbances (β not shown)	-	Age, gender, field tenure and annual salary
Ahola, Hakanen, 2007 [27]	2,555	Depressive symptoms	-	OR = 2.6 (2.0–3.5)	Sex, age and marital status
Armon et al., 2014 [37]	4,861	Depressive symptoms	-	Burnout predicted an increase in depressive symptoms from T1 to T2 (β = 0.15**)	T1 depressive symptoms, T1 neuroticism, age, gender, education, marital status, number of children, financial strain, time between T1 and T2 and chronic medical illness
Toker, Biron, 2012 [78]	1,632	Depressive symptoms	-	An increase in job burnout from T1 to T2 predicted an increase in depression from T2 to T3 (B = 0.09**)	Education in T1, depression in T2, age, gender, the time gap between T1 and T3 and visits to the medical center
Bianchi et al., 2015 [38]	627	Depressive symptoms	After adjustment for depressive symptoms at T1, burnout at T1 no longer predicted depressive symptoms at T2 (β = 0.057, p>0.05). Burnout symptoms at T1 no longer predicted cases of major depression at T2 when depressive symptoms at T1 were included in the predictive model (OR = 1.319; 0.866–2.009)	-	Gender, age, length of employment and depressive symptoms at baseline
Madsen et al., 2015 [63]	2,936	Antidepressant treatment	-	Burnout was associated with an increased risk of antidepressant treatment, particularly among men. For high versus intermediate burnout levels, burnout predicted an increased risk of antidepressant treatment of 5.17% per year of follow-up for men and 0.96% per year of follow-up for women.	Age, cohabitation, occupational position and type of organization
Leiter et al., 2013 [60]	4,356	Psychotropic and antidepressant treatment	**Antidepressants** Efficacy: β = 0.009 **Psychotropic medication** Efficacy: β = 0.009	**Antidepressants** Emotional exhaustion: β = 0.047** Cynicism: β = 0.041* Inconsistency (burnout dimensions): β = 0.051** **Psychotropic medication** Emotional exhaustion: β = 0.058** Cynicism: β = 0.038** Inconsistency (burnout dimensions): β = 0.054**	Age, sex and job characteristics
Toppinen-Tanner et al., 2009 [80]	7,897	Hospitalization for mental disorders	**Professional efficacy**—HR = 1.03 (0.88–1.20)	**Burnout**—HR = 1.37 (1.18–1.58) **Exhaustion**—HR = 1.38 (1.20–1.58) **Cynicism**—HR = 1.37 (1.18–1.57)	Age and sex, occupational status, and physical environment
De Beer et al., 2016 [44]	370	Psychological ill-health symptoms	-	**Burnout T2 (direct effect)**: β = 0.12 (0.03–0.22) **Burnout T1 (indirect effect)**: β = 0.05 (0.01–0.09)	Age and gender

*p<0.05

**p<0.01

HR = hazard ratios OR = odds ratio β = standardized partial regression coefficients B = unstandardized partial regression coefficients

Depressive symptoms were a psychological consequence that was investigated with different measurement tools, different numbers of participants and different follow-up times. Burnout was found to be a significant predictor of depressive symptoms among 2,555 dentists in a follow-up study lasting three years and a mediator between job strain and depressive symptoms [27]. In 18 months of follow-up, burnout also predicted an increase in depressive symptoms in a study of almost 5,000 apparently healthy workers attending a center for routine heath examinations in Israel [37]. Additionally, in a three-wave study conducted between the years 2003 and 2009, an increase in job burnout from T1 to T2 was found to predict an increase in depressive symptoms from T2 to T3 in 1,632 Israeli workers attending the same health center, although this increase was attenuated by high physical activity levels [78]. However, burnout did not predict depressive symptoms in a study of 627 French school teachers that did not exclude those with depressive symptoms at baseline but controlled for this outcome in the follow-up analysis [38]. In all studies selected in our review, depressive symptoms were assessed with scales typically used in epidemiological studies to screen for possible clinical depression. An increased risk of antidepressant use, mainly among men, was also demonstrated by a study [63] that used records of the Danish national prescription registry, which contains data on all prescription medications purchased at pharmacies in Denmark. Men with high levels of burnout had an increase of 5.17% per year in the risk of entering antidepressant treatment compared to men with intermediate levels of burnout; this association was stronger for men than that observed for women (an increase of 0.96% per year). Similar results were found in a Finnish study that aimed to evaluate whether changes in burnout levels over a four-year period predicted the use of psychotropic and antidepressant medications in the following eight years. The authors noted that inconsistencies among the levels of the subdimensions of burnout at baseline, such as high emotional exhaustion and low cynicism or vice versa, as well as change toward burnout in four years, were predictors of psychotropic and antidepressant treatment [60].

Burnout was also a predictor of hospital admissions due to mental disorders over a 10-year period among Finnish forest industry employees [80]. Among employees from the financial sector, burnout was a risk factor for psychological ill-health symptoms and a mediator between work overload and these symptoms [44].

### Occupational consequences

Job satisfaction, absenteeism, new disability pension, job demands, job resources and presenteeism were the investigated outcomes (Table 4). Job dissatisfaction was an occupational consequence explored in two studies with relatively small samples (n≤316). Emotional exhaustion was found to be a predictor of job dissatisfaction in both studies [47, 62], while depersonalization was found to be significantly associated in just one [47], which did not identify an association with professional efficacy.

**Table 4:** Main findings of longitudinal studies of professional consequences of burnout.

AUTHORS, YEAR	N (FINAL SAMPLE)	DEPENDENT VARIABLE	MAIN FINDINGS		
			NOT SIGNIFICANT	SIGNIFICANT	CONTROL VARIABLES
Figueiredo-Ferraz et al., 2012 [47]	316	Job satisfaction	Professional efficacy β = 0.02	Emotional exhaustion β = - 0.15** Depersonalization β = - 0.14**	Age, sex, work contract and job satisfaction in T1
Lizano, Barak, 2015 [62]	133	Job satisfaction	Depersonalization did not predict job satisfaction in low (β = 0.04) and high (β = 0.08) supervisory support groups	Higher levels of emotional exhaustion predicted lower levels of job satisfaction in both the low (β = -0.46)** and high (β = -0.48)** supervisory support groups	Age, race, tenure, position in the organization, role conflict, role ambiguity and work family
Borritz et al., 2006 [39]	824	Sickness absence days Sickness absence spells	-	**Sickness absence days** RR = 1.21 (1.11–1.32)** **Sickness absence spells** RR = 1.09 (1.02–1.17)**	Age, gender, organization status, socioeconomic status, BMI, smoking, alcohol consumption, leisure time physical activity, family status, having children below the age of 7, and diseases (diabetes, high blood pressure, chronic bronchitis, asthma, coronary thrombosis, cardiovascular spasm, cerebral hemorrhage, cerebral thrombosis, cancer, gastric ulcer, cystitis, menstruation-related pain, mental disorder, allergy, skin diseases, and backache)
Schaufeli et al., 2009 [71]	201	Absence duration	-	T1 burnout predicts T1–T2 absence duration (β = 0.26)	Age was not controlled for, but the authors reported that no significant correlations were observed between age and any of the study variables
Borritz et al., 2010 [40]	1,734	Long‑term sickness absence (>2 weeks)	-	**Highest level of work burnout Model 1**: RR = 2.93 (1.89–3.96) **Model 2**: RR = 2.67 (1.79–3.55) **Model 3**: RR = 2.67 (1.80–3.55) **Model 4**: RR = 2.81 (1.89–3.72) **Model 5**: RR = 2.72 (1.83–3.60) **Model 6**: RR = 2.77 (1.87–3.67) **Medium level of work burnout Model 1**: RR = 1.70 (1.11–2.29) **Model 2**: RR = 1.57 (1.06–2.08) **Model 3**: RR = 1.54 (1.05–2.04) **Model 4**: RR = 1.58 (1.07–2.09) **Model 5**: RR = 1.56 (1.06–2.07) **Model 6**: RR = 1.57 (1.06–2.07)	**Model 1**: age, gender, socioeconomic status, family status, health-related lifestyle (smoking habits, alcohol consumption, sedentary lifestyle, overweight, underweight, and presence of chronic physical disease) **Model 2**: Model 1 plus emotional demands **Model 3**: Model 1 plus role conflicts **Model 4**: Model 1 plus role clarity **Model 5**: Model 1 plus predictability **Model 6**: Model 1 plus quality of leadership
Hallsten et al., 2011 [51]	4,109	Long‑term sickness absence (>60 consecutive days)	-	OR = 2.05 (1.13–3.70)	Gender, age group, level of occupational skill, family status, chronic disorders, daily smoking and previous sickness absence
Roelen et al., 2015 [68]	4,894	Long‑term sickness absence(≥42 consecutive days)	**Musculoskeletal long-term sickness absence** OR = 1.38 (0.74–2.58)	**Mental long-term sickness absence** OR = 1.55 (1.07–2.25)*	Age, gender, marital status, children at home, employment, work hours/week, tenure in work, BMI, physical activity, smoking habits, alcohol consumption and the use of drugs and sedatives
Toppinen-Tanner et al., 2005 [79]	3,895	Sick-Leave absences (≥3 days absence episodes, medically certified)	**HIGH BURNOUT Sick-leave absences due to diseases of the digestive system** RR = 1.65 (0.86–3.18)	**HIGH BURNOUT Sick-leave absences due to mental and behavioral disorders** RR = 3.15 (1.38–7.19) **Sick-leave absences due to diseases of the circulatory system** RR = 1.89 (1.00–3.60) **Sick-leave absences due to diseases of the respiratory system** RR = 1.29 (1.04–1.61) **Sick-leave absences due to diseases of the musculoskeletal system** RR = 1.26 (1.04–1.52)	Age, gender and employee group
Ahola et al., 2009 [28]	3,125	New disability pension	-	OR = 1.49 (1.24–1.80)	Gender, age, marital status, occupational status, occupational sector, mental disorders, and physical illnesses
Ahola et al., 2009 [29]	7,810	New disability pension	**Mild burnout** HR = 1.16 (0.96–1.39)	**Severe burnout** HR = 1.57 (1.09–2.26)	Gender, age, marital status, socioeconomic status, registered medication use and self-reported chronic illness
Wang et al., 2016 [83]	263	Job demands and job resources	T1 job burnout did not affect T2 job demands (r = -0.11)	T1 job burnout affected T2 job resources (r = 0.09)* T2 job burnout affected job resources (r = -0.14)* and job demands at T3 (r = 0.15)*	Age, organizational tenure, marital status, gender, level of education, and managerial status
Demerouti et al., 2009 [45]	258	Job demands and presenteeism	T1 depersonalization did not lead to more presenteeism	T1 emotional exhaustion had effects on both T2 and T3 presenteeism. T1 emotional exhaustion and depersonalization had significant effects on T2 job demands. T1 depersonalization had an additional effect on T3 job demands	Gender and general heath in T1

*p<0.05

**p<0.01

HR = hazard ratios OR = odds ratio RR = risk ratio r = synchronous correlations (within-wave correlations between the errors) β = standardized partial regression coefficients

Burnout was prospectively associated with sickness absence days and sickness absence spells. Workers with worse levels of burnout (those ranked in the highest quartile of the scale score) were absent from work, on average, 13.6 days per year, in comparison with those classified in the lowest quartile (5.4 days). An increase in the burnout score predicted increases of 21% and 9% in sickness absence days and sickness absence spells, respectively, even after adjustments for sociodemographic, work and health conditions [39]. An increase in absence duration (defined as the number of sick-leave days between T1 and T2) was an occupational consequence among workers with high levels of burnout [71].

The high and medium levels of burnout were associated with long-term sickness absence (>2 weeks) after adjustments for sociodemographic characteristics and health-related lifestyle. When psychosocial characteristics were added in the statistical model for adjustments, the effects of high and medium levels of work burnout were attenuated, although the results were still significant [40].

In a study that grouped the exhaustion and cynicism dimensions of burnout (ExCy), Hallsten et al. [51] observed a twofold risk of long-term sickness absence (>60 consecutive days) among those classified as high ExCy after adjustments. Burnout was also a predictor of both long-term sickness absence leaves (≥42 consecutive days) [68] and sick-leave absences (≥3 days) due to mental or behavioral problems [79]. Sick leaves due to musculoskeletal disorders of ≥3 days were a consequence of burnout [79], but no association was identified for long-term sickness absences due to these disorders (≥42 consecutive days) [68]. Additionally, burnout was a significant risk factor for sick-leave absences due to diseases of the circulatory and respiratory systems but not for diseases of the digestive system [79].

A study of 3,125 Finnish forest industry employees found that burnout significantly predicted new disability pensions during a 4-year follow-up period, even after adjustments for age, gender, marital status, occupational status, sector, mental disorders and physical illness. The same pattern of association was found, after all adjustments, for the burnout syndrome subdimensions of cynicism (in both genders) and emotional exhaustion (in men) [28]. These investigators, in a subsequent follow-up study [29] that lasted eight years and included 7,810 employees of the same forest industry, found that workers with severe burnout had a greater likelihood of receiving a new disability pension (15%) compared to those with mild (8%) or no burnout (5%). After adjustments for several confounders and chronic illness at baseline, severe burnout and severe emotional exhaustion significantly predicted a new disability pension. However, when analyzing causes of disabilities according to the presence of burnout and its subdimensions, only exhaustion significantly predicted new pensions for mental or other miscellaneous diseases, after all adjustments [29].

Job burnout prospectively affected the perception of job demands and job resources among Chinese workers [83]. A study of nurses revealed that both emotional exhaustion and depersonalization had effects on future perception of high job demands and that emotional exhaustion predicted presenteeism [45].

**Fig 3:**
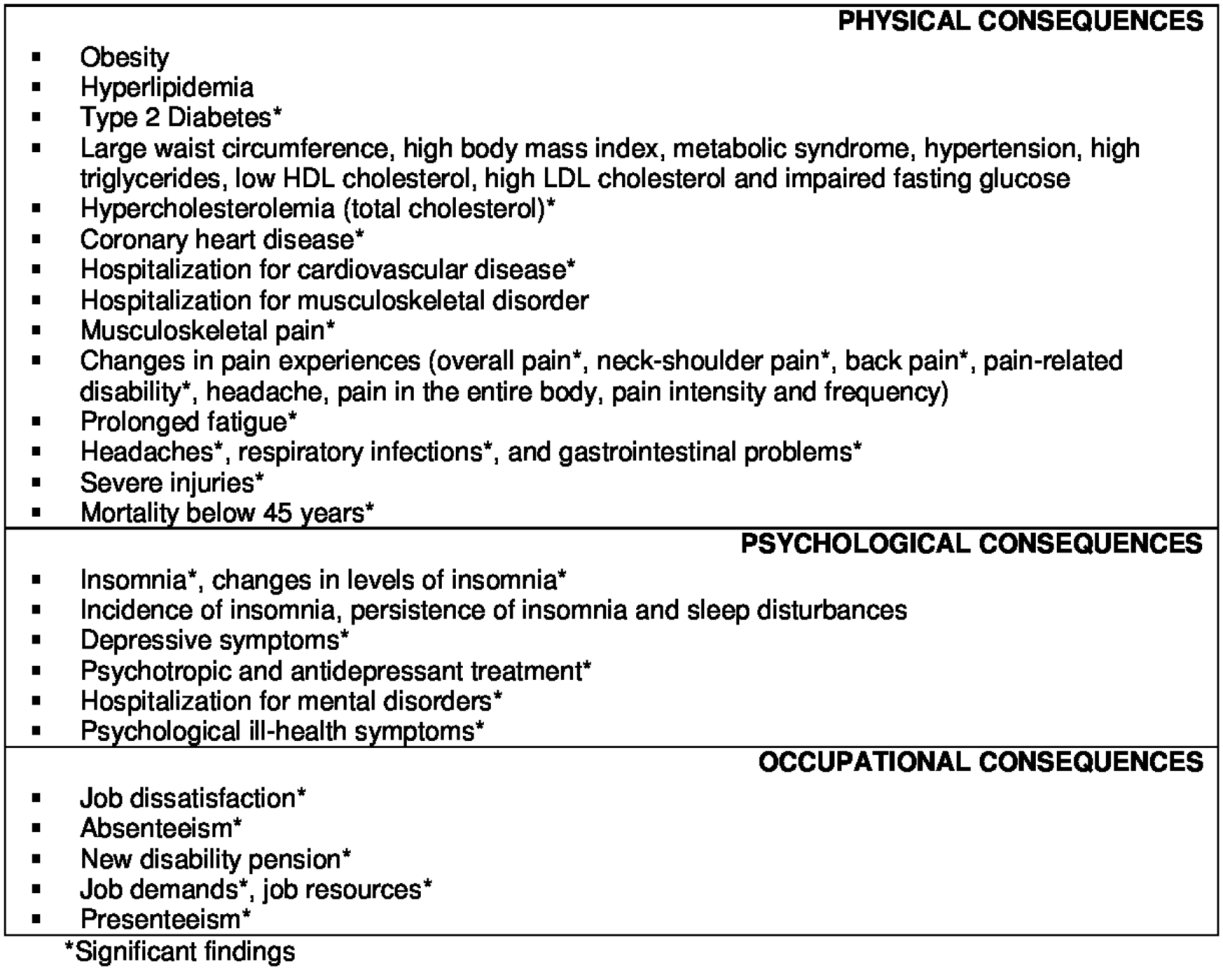
Physical, psychological and occupational consequences of burnout investigated in prospective studies with better methodological quality.

The investigated and statistically significant consequences of burnout are shown in Fig 3.

## Discussion

To the best of our knowledge, this is the first comprehensive systematic review of the prospective effects of job burnout. This review provides relevant evidence of the physical, psychological and occupational consequences of this syndrome to workers. No limits were defined for the literature search, such as language or time of publication. Selection and methodological evaluation of the articles were performed independently by two authors and followed PRISMA guidelines [12]. To guarantee a higher quality of evidence, we defined minimal criteria that should be followed when prospective studies are conducted in order to avoid selection bias or other types of bias [13, 19, 23–26]. Nevertheless, a meta-analysis could not be performed due to the heterogeneity of the studies, mainly regarding measures of burnout or outcomes that were analyzed in more than one study (e.g., depressive symptoms or absenteeism). Therefore, we were not able to assess publication bias with statistical procedures, because the results of the included articles could not be analyzed using meta-analysis methods. We cannot rule out the possibility that some of the excluded studies are also of high methodological quality but were not included because they did not report exclusion of the outcome at baseline or they failed to report adjustments. Finally, studies with students were not included in our review, although undergraduate and graduate medical students in particular may experience many of the stressors and consequences of professional burnout [88]. We found only one study that longitudinally analyzed burnout as a potential risk factor for ill health among medical students; burnout (depersonalization and a low sense of personal accomplishment) was a predictor of suicidal intention over the following one year, whereas recovery from burnout reduced the risk of this outcome [88]. This serious and life-threatening outcome may also occur in working populations experiencing burnout.

The majority of the cohort studies selected for this review was from Nordic countries. This may be due to the availability of reliable registries on health and social benefits in these countries [8, 28–31, 40, 63, 68], which make it possible to identify employees, link records and therefore conduct studies of large sample sizes. It is important to note that the political and economic situation in these countries favors better work and health conditions. Therefore, there is still room for research in low- and middle-income countries, where work conditions and access to healthcare are less favorable. In such regions, for instance, workers may not have the option of choosing their job or may not be able to quit their job for survival reasons. In addition, low- and middle-income countries generally do not have well-structured health services or state-of-the-art technologies, and the quality of assistance may be less than optimal, all of which can affect the health of the population.

Among the physical consequences of burnout that were prospectively investigated, cardiovascular diseases and pain stood out. Cardiovascular diseases were also more frequently reported as causes of absenteeism by workers with burnout in a study classified under occupational consequences. As burnout follows a state of chronic stress, it has been suggested that the biological mechanisms resulting from prolonged stress may deteriorate physical health. One hypothesis is that the autonomic nervous system (ANS) and the hypothalamic-pituitary-adrenal (HPA) axis become exhausted due to burnout. This results in overactivation of vital functions (e.g., heart rate and blood pressure) and damage to metabolism and the immune system [89]. According to Melamed et al. [90], potential mechanisms linking burnout to cardiovascular diseases include its associations with components of metabolic syndrome, dysregulation of the HPA axis, inflammation, sleep disorders, reduced immunity, changes in blood coagulation, changes in fibrinolysis, and adoption of poor health behaviors, such as smoking and lack of physical activity. In fact, cumulative work stress has been shown to be associated with the incidence of cardiovascular events, and this relationship was mediated by both the direct effects of neuroendocrine mechanisms and the indirect effects of unhealthy behaviors, particularly poor diet and low physical activity [91].

Impaired immune function in individuals with burnout may increase their susceptibility to infectious diseases such as flu-like illnesses, the common cold and gastroenteritis [67], which is in line with the results observed for respiratory infections and gastroenteritis in one study included in this review [58]. In addition, burnout was a predictor of musculoskeletal pain, although the pathophysiological pathways linking these conditions remain unclear [66]. The findings regarding the longitudinal relationship between burnout and headaches were not consistent, one study detected a significant association [58], whereas the other study did not [48]. This divergence may be due to the different methodologies used and may be mainly related to the previous timeframe in which headache was investigated (six months vs three months) and the definition of headache used, among other factors. Moreover, the type of headache was not investigated (tension headache, acute or chronic headache, migraine) in either study. In epidemiological studies, the case definition of headache can be problematic [92], particularly tension-type headaches, which are the most common type. This issue may result in different rates of incidence or prevalence [92] and can impact on the investigated associations. However, there is evidence that psychological stress, which is closely related to burnout and pain, is a contributing factor to headache [93], and several mechanisms linking stress to headache have been proposed, such as sympathetic hyperactivity [94]. Workers under recurrent or prolonged stress may also more frequently engage in unhealthy behaviors, such as a poor or rich diet, a lack of physical exercise and alcohol abuse. When combined with sleep disorders, these unhealthy behaviors may lead to a myriad of other consequences [35, 59], such as obesity [8] and diabetes [65].

Burnout significantly predicted depressive symptoms or antidepressant treatment in the majority of the studies that investigated psychological consequences, and these relationships were stronger for the subdimensions emotional exhaustion and depersonalization. Some authors have recently renewed debate on the overlap between burnout and depression, as they found that these conditions were highly correlated with each other, therefore advocating that burnout should be considered a depressive syndrome [95]. However, Maslach and Leiter [4] have argued that there are problems with this analysis since the instruments used for measuring burnout and depression are both dominated by fatigue. They concluded that it is not surprising that they would overlap. Moreover, studies on the discriminant validity of burnout and depression [96, 97] have demonstrated that these conditions are distinct constructs. In the present review, studies that detected associations between burnout and depressive symptoms either excluded those affected by this outcome at baseline or controlled for these symptoms, which favors the argument that these are indeed distinct conditions.

The results regarding the incidence of sleep problems following burnout were not consistent in our review. Insomnia or changes in insomnia levels were found to be consequences of burnout in two studies with apparently healthy employees attending a center for periodic health examinations in Israel [33, 35] but not in a study with a random sample of employed individuals taken from the general population of the Örebro County, Sweden [55]. Another study with social workers in the state of California, USA, also did not find that burnout was a risk factor for sleep disturbances, although the final sample of this study was relatively small (n = 146) [58]. These differences may be due to the varying methodologies of the studies, with different tools used to measure burnout or sleep disturbances. Moreover, the studies with Israeli workers excluded people with cardiovascular diseases, diabetes, those who had suffered a stroke or a mental crisis and those taking antidepressants or antipsychotic medication [33, 35], which are conditions usually related to burnout or sleep problems and thus increased internal validity. Conversely, the Swedish study included employees regardless of their health statuses, perhaps thereby increasing external validity, although reducing internal validity [55]. More longitudinal studies with both internal and external validity are needed to explore the incidence of sleep problems among burned out workers. Measuring sleep disturbances with objective measures, such as actigraphy or polysomnography, is also important [98].

Burnout can also trigger presenteeism [45] and absenteeism [39, 40, 51, 68, 79]. While absenteeism means absence from work, presenteeism represents a phenomenon when people come to work even when sick, leading to a loss of productivity. In a vicious circle, as a consequence of health problems caused by burnout, workers may not reach the desirable performance at work, which in turn may lead to increasing levels of emotional exhaustion [86, 99]. The worker’s weakened health along with his/her diminished functional capacity may lead to absenteeism, a great cause of concern for the worker and the organizations that has both social and economic consequences. For the individual, absenteeism or presenteeism due to health problems may represent the beginning of a process of social decline involving job loss and even permanent exclusion from the labor market. For organizations, absenteeism means a loss of manpower, additional expenses associated with temporary workers and a decrease in productivity [39]. In this review, we observed that workers who experienced medium or high levels of burnout were at higher risk of short or long-term sickness absences. In addition to absenteeism, there is evidence that burnout also increased the risk of future disability pension [28, 29].

In our review, we focused on the longitudinal relationships between burnout and physical, psychological and occupational outcomes and we highlighted some of the complex mechanisms involved in this process. Some of these mechanisms have been investigated in several studies on the antecedents and outcomes of burnout, most of which have adopted the theoretical framework of the job demands-resources (JD-R) model [100]. This model posits that work demands (e.g., high workload and time pressure) leads to negative outcomes via burnout (stress process), whereas work resources (e.g., autonomy and peers’ support) via work engagement contribute to positive outcomes (motivational process) [101, 102]. Studies have shown that burnout is more stable than engagement over time [103, 104]. More recently, leadership was integrated into the JD-R model to examine its relationships with a variety of demands, resources and outcomes. The authors found that leadership had a direct effect on the investigated outcomes (employability, performance, commitment and performance behavior) and an indirect effect on burnout and engagement by reducing demands and increasing job resources [102]. Reports have suggested that both the level of demands and the types of demands (challenge or hindrance) can impact the work-related well-being of employees, as revealed in a study in which job resources (support from colleagues, performance feedback, supervisor coaching and opportunities for development) fostered workers’ well-being (positive affectivity and work engagement) specifically under high-challenge demand conditions but not under hindrance demands [105]. There is also an ongoing debate about the concept of engagement regarding its relationship with burnout (i.e., if engagement is the opposite of burnout or if they are distinct constructs) [4, 106]. In a recent meta-analytic study, Goering et al. [106] showed that burnout and engagement seemed to be distinct constructs when antecedents (resources, challenge demands and hindrance demands) were analyzed; however, the pattern was less clear for consequences (performance, turnover intention, job satisfaction, organization commitment and indicators of physical health). Burnout and engagement seem to act as opposites for turnover intention and task performance; however, due to the large heterogeneity of effect sizes in the population with distributions ranging from positive to negative values, the authors concluded that several boundary conditions (moderators) might exist, many of which probably had not been investigated to date. These aforementioned aspects in connection with the results of our review suggest the need to more deeply investigate the process leading from burnout to health or occupational outcomes, especially by incorporating factors that may confound the relationship and analyzing the role of possible mediators that can change the pattern of associations (e.g., moderating the relationships). As shown in our review, the majority of studies had only two waves. Considering the dynamics and the complexity of the relationship between variables, knowledge could be extended by studies with multiple waves to try to capture changes in work conditions, burnout, engagement and the incidence of diverse outcomes [27, 44, 49].

In summary, the present systematic review based on well-conducted and well-reported studies shows that cardiovascular diseases, musculoskeletal pain, depressive symptoms, psychotropic and antidepressant treatment, job dissatisfaction and absenteeism are consistent effects of burnout. Conflicting findings were observed for headache and insomnia. Other consequences were found in only one study; therefore, there is still a need to investigate these relationships with burnout in longitudinal studies. The individual and social impacts of burnout highlight the need for preventive interventions and early identification of this health condition in the work environment.

## Supporting information

S1 Appendix Prisma checklist. (DOC)

S2 Appendix Search strategy. (DOCX)

S3 Appendix Assessment of quality in cohort studies about burnout consequences. (DOCX)

S4 Appendix Characteristics of the 25 articles not included in this systematic review. (DOCX)
