# Supplementary material for: Physical, psychological and occupational consequences of job burnout: A systematic review of prospective studies
Source: PLoS One. 2017 Oct 4;12(10):e0185781. doi: 10.1371/journal.pone.0185781 (PMC5627926; doi:10.1371/journal.pone.0185781)
Supplement: S2 Appendix — (DOCX) [file pone.0185781.s002.docx]

**Search Strategy**

1. Pubmed - ((burnout[Title]) AND (longitudinal*[Title/Abstract] OR prospective[Title/Abstract] OR cohort[Title/Abstract] OR case control[Title/Abstract] OR case-control[Title/Abstract] or "follow-up"[Title/Abstract] or "follow up"[Title/Abstract]))
2. Science Direct - TITLE(burnout) and TITLE-ABSTR-KEY(longitudinal* OR prospective OR cohort OR case control OR case-control OR follow-up OR "follow up").
3. PsycInfo -Title: burnout AND Abstract: longitudinal* OR prospective OR cohort OR case control OR case-control OR follow-up OR follow up
4. Lilacs - burnout [Title] and longitudinal$ OR prospective OR cohort OR case control OR case-control OR follow up OR follow-up [Abstract]
5. Scielo - (ti:(burnout)) AND (ab:(longitudinal* OR prospective OR cohort OR case control OR case-control OR follow up OR follow-up))
6. Web of Science - TI=(burnout) AND TS=(longitudinal* OR prospective OR cohort OR case control OR case-control OR follow up OR follow-up)
