# Supplementary material for: Physical, psychological and occupational consequences of job burnout: A systematic review of prospective studies
Source: PLoS One. 2017 Oct 4;12(10):e0185781. doi: 10.1371/journal.pone.0185781 (PMC5627926; doi:10.1371/journal.pone.0185781)
Supplement: S3 Appendix — (DOCX) [file pone.0185781.s003.docx]

| **AUTHORS, YEAR** | **Outcome was investigated at baseline and was excluded or controlled for in the follow-up analysis** | **Follow-up rate >50% and/or non-response analysis was presented** | **Adjusted for age or health conditions** | **Analyzed in this systematic review?** |
| --- | --- | --- | --- | --- |
| Ahola, Hakanen, 2007 [27] | + | + | + | + |
| Ahola et al., 2009 [28] | + | + | + | + |
| Ahola et al., 2009 [29] | + | + | + | + |
| Ahola et al., 2010 [30] | + | + | + | + |
| Ahola et al., 2013 [31] | + | + | + | + |
| Appels, Schouten, 1991 [32] | + | + | + | + |
| Armon et al., 2008 [33] | + | + | + | + |
| Armon et al., 2008 [34] | + | + | + | + |
| Armon, 2009 [35] | + | + | + | + |
| Armon et al., 2010 [36] | + | + | + | + |
| Armon et al., 2014 [37] | + | + | + | + |
| Bianchi et al., 2015 [38] | + | + | + | + |
| Borritz et al., 2006 [39] | + | + | + | + |
| Borritz et al., 2010 [40] | + | + | + | + |
| Burke, Greenglass,1995 [41] | - | + | + | - |
| Burke et al., 1996 [42] | - | + | - | - |
| De Beer et al., 2013 [43] | + | + | - | - |
| De Beer et al., 2016 [44] | + | + | + | + |
| Demerouti et al., 2009 [45] | + | + | + | + |
| Dubois et al., 2014 [46] | + | + | - | - |
| Figueiredo-Ferraz et al., 2012 [47] | + | + | + | + |
| Grossi et al., 2009 [48] | + | + | + | + |
| Hakanen et al., 2008 [49] | + | + | - | - |
| Hakanen, Schaufeli, 2012 [50] | + | + | - | - |
| Hallsten et al., 2011 [51] | + | + | + | + |
| Huang et al., 2016 [52] | + | + | - | - |
| Idris et al., 2014 [53] | + | + | - | - |
| Innstrand et al., 2008 [54] | + | + | - | - |
| Jansson-Fröjmark, Lindblom, 2010 [55] | + | + | + | + |
| Kadzielski et al., 2012 [56] | - | + | + | - |
| Khamisa et al., 2016 [57] | - | - | - | - |
| Kim et al., 2011 [58] | + | + | + | + |
| Kitaoka-Higashiguchi et al., 2009 [59] | + | + | + | + |
| Leiter et al., 2013 [60] | + | + | + | + |
| Leone et al., 2009 [61] | + | + | + | + |
| Lizano, Barak, 2015 [62] | + | + | + | + |
| Madsen et al., 2015 [63] | + | + | + | + |
| McManus et al., 2002 [64] | + | + | - | - |
| Melamed et al., 2006 [65] | + | + | + | + |
| Melamed, 2009 [66] | + | + | + | + |
| Mohren et al., 2003 [67] | + | - | + | - |
| Roelen et al., 2015 [68] | + | + | + | + |
| Rudman, Gustavsson, 2011 [69] | + | + | - | - |
| Rudman, Gustavsson, 2012 [70] | + | + | - | - |
| Schaufeli et al., 2009 [71] | + | + | + | + |
| Shin et al., 2013 [72] | - | - | - | - |
| Shirom et al., 1997 [73] | + | - | + | - |
| Shirom et al., 2013 [74] | + | + | + | + |
| Shoji et al., 2015 [75] | + | + | - | - |
| Tang et al., 2001 [76] | - | + | + | - |
| Toker et al., 2012 [77] | + | + | + | + |
| Toker, Biron, 2012 [78] | + | + | + | + |
| Toppinen-Tanner et al., 2005 [79] | + | + | + | + |
| Toppinen-Tanner et al., 2009 [80] | + | + | + | + |
| Travis et al., 2015 [81] | - | + | + | - |
| Vinokur et al., 2009 [82] | + | + | - | - |
| Wang et al., 2016 [83] | + | + | + | + |
| Wolpin et al., 1991 [84] | - | + | - | - |
| Wright, Bonett, 1997 [85] | + | - | + | - |
| Wright, Cropanzano, 1998 [86] | + | + | - | - |
| Ybema et al., 2010 [87] | + | + | - | - |

(+) Yes, (-) No
