# Supplementary material for: Physical, psychological and occupational consequences of job burnout: A systematic review of prospective studies
Source: PLoS One. 2017 Oct 4;12(10):e0185781. doi: 10.1371/journal.pone.0185781 (PMC5627926; doi:10.1371/journal.pone.0185781)
Supplement: S4 Appendix — (DOCX) [file pone.0185781.s004.docx]

| **AUTHORS, YEAR**  **COUNTRY** | **POPULATION**  **(FINAL SAMPLE)** | **FOLLOW-UP**  **PERIOD** | **BURNOUT INVENTORY** | **BURNOUT DATA** | **DEPENDENT VARIABLE** |
| --- | --- | --- | --- | --- | --- |
| Burke, Greenglass, 1995  Canada [41] | 362 school-based educators  (teachers and administrators) | 1 year | Maslach Burnout Inventory (MBI) | Continuous variable (score of each dimension of burnout; total score MBI) | Psychosomatic symptoms (e.g., headaches, poor appetite, feeling lonely)  General medication use (e.g., tranquilizers, sleeping pills, pain medication) |
| Burke et al., 1996  Canada [42] | 362 school-based educators  (teachers and administrators) | 1 year | MBI | Continuous variable (score of each dimension of burnout; total score MBI) | Weakness, heart symptoms and depressive mood |
| De Beer et al., 2013  South Africa [43] | 593 employees from a mining organization | 1 year | Burnout was measured  and constructed by two core components: exhaustion and  mental distance (cynicism) | Continuous variable (mean score of burnout) | Job resources and work overload |
| Dubois et al., 2014  Canada [46] | 96 employees that included registered nurses, assistant nurses, rehabilitation workers and psychosocial workers | 1 year | The nine-item Emotional Exhaustion subscale of MBI | Continuous variable (mean score of emotional exhaustion) | Commitment to change and cynicism toward change in management |
| Hakanen et al., 2008  Finland [49] | 2,555 dentists | 3 years | MBI | Continuous variable (emotional exhaustion and depersonalization) | Depressive symptoms |
| Hakanen, Schaufeli, 2012  Finland [50] | 1,964 dentists | 7 years  T1-T2: 3 years, T2-T3: 4 years | MBI | Continuous variable (mean scores of emotional exhaustion and depersonalization) | Depressive symptoms |
| Huang et al., 2016  China [52] | 104 leader-follower dyads of software development, electronic engineering and agricultural products | 6 months | MBI | Continuous variable (score of each dimension of burnout) | Follower burnout |
| Idris et al., 2014  Malaysia [49] | 117 employees of private sector organizations | 3 months | MBI | Continuous variable (emotional exhaustion subscale) | Depression |
| Innstrand et al., 2008  Norway [54] | 2,235 respondents from eight different occupational groups (lawyers, bus drivers, employees within information technology, physicians, teachers, church ministers, employees within advertisement, and nurses) | 2 years (two points in time with a 2-year time interval) | Oldenburg Burnout Inventory (OLBI) | Continuous variable (mean scores of emotional exhaustion and disengagement) | Work family conflict |
| Kadzielski et al., 2012  USA [56] | 51 employed, English-speaking, adult patients with isolated finger injuries | 0.5 year | Shirom-Melamed Burnout Measure (SMBM) | Continuous variable (mean of burnout score) | Pain or arm-specific disability |
| Khamisa et al., 2016  South Africa [57] | 277 nurses from four hospitals | 1 year | MBI | Categorized as high, moderate and low for each subscale (emotional exhaustion, depersonalization and personal accomplishment) | Job satisfaction and general health |
| McManus et al., 2002  United Kingston [64] | 331 doctors | 3 years | MBI | Continuous variable (mean scores of emotional exhaustion, depersonalization and personal accomplishment) | Stress |
| Mohren et al., 2003  Netherlands [67] | Employees from 45 different companies and organizations (Maastricht Cohort Study)  N follow-up not identified | 3 years (annually) | MBI | **Clinical burnout:** scoring in the 95th percentile of the Exhaustion and Cynicism scales or in the 5th percentile of the professional efficacy scale  **Probable burnout:** Subjects scoring in the upper quartile of the Exhaustion and Cynicism scales or in the bottom quartile of the professional efficacy scale | Common infections: common cold, flu-like illness and gastroenteritis |
| Rudman, Gustavsson 2011  Sweden [69] | 997 nurses (Population-based Longitudinal Analysis of Nursing Education/Entry study) | 3 years (annually) | OLBI | Summative scale, with ranged between 6 and 24. Burnout symptoms were classified as: low (6-11.9), moderate (12-18), high (18.1-24) burnout | Depressive mood and intention to leave the profession |
| Rudman, Gustavsson, 2012  Sweden [70] | 1,401 nurses (Population-based Longitudinal Analysis of Nursing Education/Entry study) | 4 years (annually) | OLBI | Continuous variable (mean score of emotional exhaustion and disengagement) | Depressive symptoms |
| Shin et al., 2013  South Korea [72] | 499 middle and high school teachers | 1.5 years (every 6 months) | MBI | Continuous variable (mean of burnout score) | Depression |
| Shirom et al., 1997  Israel [73] | 665 apparently healthy employees at periodic health examinations in a health-screening center | 2-3 years | Emotional burnout (5 items) by Shirom | Continuous variable (mean score of emotional burnout) | Cholesterol and  triglycerides levels |
| Shoji et al., 2015  Poland [75] | Study 1: 135 behavioral and mental healthcare providers working with U.S. military personnel  Study 2: 194 healthcare and social workers providing services for civilians who had experienced traumatic events | Study 1: mean 195.80 days (SD = 20.00)  Study 2: mean 162.35 days (SD = 39.51) | OLBI | Continuous variable (mean scores of job burnout, emotional exhaustion and disengagement) | Secondary traumatic stress |
| Tang et al., 2001  Hong Kong [76] | 61 secondary school teachers  (Study 2) | 0.5 year | MBI | Continuous variable (mean scores of emotional exhaustion, depersonalization and lack of personal accomplishment) | Negative mental health |
| Travis et al., 2015  USA [81] | 362 employees at a large urban public child welfare agency | 1 year (at three six-month intervals) | MBI | Continuous variable (mean score of emotional exhaustion and depersonalization) | Work withdrawal and exit-seeking behaviours |
| Vinokur et al., 2009  USA [82] | 1,009 air force personnel deployed to the wars in Iraq, Afghanistan and supporting locations | 1 year | SMBM | Continuous variable (mean scores of job burnout, physical fatigue, emotional exhaustion, depersonalization and cognitive weariness) | Decrease in perceived health |
| Wolpin et al., 1991  Country not identified [84] | 245 teachers | 1 year | MBI | Continuous variable (mean scores of emotional exhaustion, depersonalization and lack of personal accomplishment) | Job satisfaction |
| Wright, Bonett, 1997  USA [85] | 44 employees of a public human services department | 3 years | MBI | Continuous variable (mean scores of emotional exhaustion, depersonalization and diminished personal accomplishment) | Work performance |
| Wright, Cropanzano, 1998  USA [86] | 52 social welfare workers | 1 year | MBI | Continuous variable (mean score of emotional exhaustion) | Job performance  and voluntary turnover |
| Ybema et al., 2010  Netherlands [87] | 844 employees of 34 companies | 4 years | Utrecht Burnout Scale | Continuous variable (mean of burnout score) | Time lost and job satisfaction |
